# Attitudes, beliefs and knowledge about medical cannabis among nurses and midwives in Cyprus: a cross-sectional descriptive correlational study

**DOI:** 10.1186/s12912-022-00887-1

**Published:** 2022-05-19

**Authors:** S. Sokratous, K. Kaikoush, M. D. Mpouzika, G. Alexandrou, N. M. Karanikola

**Affiliations:** 1 Assistant Professor, Department of Nursing, School of Health Sciences, Cyprus University of Technology, Vragadinou Street, Limassol, Cyprus; 2 PhD, Mental health Nurse, Cyprus Mental Health Services, Larnaca, Cyprus; 3 Assistant Professor, Critical Care Nursing, Advanced Emergency and Intensive Nursing Care, Department of Nursing, School of Health Sciences, Cyprus University of Technology, Vragadinou Street, Limassol, Cyprus; 4 PhD(c), Mental health Nurse, Cyprus Mental Health Services, Nicosia, Cyprus; 5 Associate Professor, Department of Nursing, School of Health Sciences, Faculty of Health Sciences, Mental Health Studies & Research Cyprus University of Technology Chair Cyprus University of Technology Vragadinou Street, Limassol, Cyprus

**Keywords:** Cypriot nurses, Midwives, Attitudes, Beliefs, Knowledge, Medical cannabis

## Abstract

**Background:**

There is a lack of evidence on healthcare professionals’ attitudes, knowledge, and beliefs about medical cannabis in Cyprus and across the world. Therefore, the present study aimed to explore the attitudes, beliefs, and knowledge about MC use among nurses and midwives in Cyprus. Special focus was given to differences across gender, age, religion, marital status, and years of work experience.

**Methods:**

A descriptive, cross-sectional correlational study with internal comparisons was conducted during the 26^th^ Nurses and Midwives Congress in Cyprus. All active nurses and midwives (convenience sampling), from the private and national healthcare services (*n* = 526) were eligible to participate. To analyze the data, the Pearson Chi-square test for group differences was employed, and descriptive and inferential statistics were assessed.

**Results:**

The final sample population consisted of 232 nurses and midwives (response rate of 46.4%). In total, 67(28.9%) participants were male, and 165(71.1%) were female. Cypriot nurses and midwives reported lack of knowledge regarding the risks and benefits about MC use to patients. However, specific number of participants believed MC use was considered acceptable for the patients with persistent muscle spasms, insomnia/sleeping disorders, mental health conditions, and terminal illnesses. The vast majority of the participants believed that formal training on MC should be integrated into academic programs, and expressed the necessity of urgent training under the current curriculum, as well as, educational training programs about MC use should be integrated into the practice/clinical practice. Concerning the socio-demographic characteristics of the participants, gender had a statistically significant positive effect on participants’ attitudes and beliefs about MC (*p* < 0.01, 26.8% vs. 13.4%). Male and unmarried participants reported higher frequency about cannabis use for recreational purposes, compared with female group (*p* < 0.01, 22.8%Vs 11.4%). Unmarried participants agreed that using cannabis might develop serious mental health risks compared with married participants group (*p* < 0.05, 77.9% vs. 66.8%).

**Conclusions:**

The conclusions seem to be rather recommending in favor of MC use**.** Participants proposed enriching nursing curricula with theoretical and clinical/laboratory courses about MC during studies and clinical practice. Additional tailoring interventions should be established to decrease recreational cannabis use among Cypriot nurses and midwives.

## Background

The term cannabis refers to pharmacologic agents derived from plants belonging to the genus Cannabis [1]. Cannabidiol (CBD), a cannabis compound, is associated with many therapeutic effects [2]. Healthcare providers have the authority to recommend non-pharmaceutical CBD products for therapeutic purposes, in compliance with state law [3]. In its comprehensive review of the medical literature on the health effects of cannabis and cannabinoids [4], the National Academies of Sciences, Engineering, and Medicine (NASEM) found substantial evidence of the effectiveness of medical cannabis (MC) in alleviating chronic pain, chemotherapy-induced nausea and vomiting, and spasticity associated with multiple sclerosis (MS) [5].

Evidence from the literature supported that MC effectively reduced patients’ chronic disease symptoms, such as chronic pain and their use of opioid medication for pain management [6]. Furthermore, research evidence pointed out, that they are positive effect between MC use and anxiety symptoms. Specifically, the patients reported that the MC use helped them to manage their anxiety symptoms [7]. Finally, qualifying conditions for MC in Minnesota currently include cancer associated with severe/chronic pain, nausea/severe vomiting, and/or cachexia/severe wasting; glaucoma; HIV/AIDS; Tourette syndrome; amyotrophic lateral sclerosis (ALS); seizures, epilepsy; severe and persistent muscle spasms, including those characteristic of MS; inflammatory bowel disease, including Crohn’s disease; terminal illness with a probable life expectancy of less than one year; intractable pain; post-traumatic stress disorder; obstructive sleep apnea; and autism [8].

There are varieties in the cannabis products used in clinical practice, as a result these variations have created uncertainty and reservations for healthcare providers attempting to make decisions and educate patients about MC [9]. Furthermore, there is little evidence on the attitudes, knowledge, and beliefs of healthcare professionals and students toward MC in Cyprus and across the world [10]. In the study of Philpot et. Al. [9], the authors examine the primary care providers’ beliefs and attitudes about MC use and its effects on medical conditions in they are clinical practice. The results show that more of the half of the participants believed that MC is involve positively in the treatment of specific medical conditions such as cancer, terminal illness, and intractable pain [9]. However, half of the participants in the same study reported that they did not have the appropriate theoretical background to answer patient’s questions about MC, and the majority of them wanted to learn more about MC use and its benefits for medical conditions [9]. Additionally, other studies have highlighted the lack of knowledge among healthcare professionals about MC use as well as the lack of clinical practice guidance on the topic [11].

Finally concerning Republic of Cyprus, MC was legalized for medical use during the 2010s. The legislation mainly focused on producers and specified licensing procedures, authorized activities, safety measures, and good production practices [12]. It also mandated that medical prescriptions include the type of hemp, the trade name, the percentage of Delta-9-Tetrahydrocannabinol and CBD content, the route of administration, the dosage, and the total monthly quantity as well as physician and patient contact details [12].

### Aim

The present study aimed to explore the attitudes, beliefs, and knowledge of nurses and midwives in Cyprus about MC use. Special focus was given to differences across gender, age, religion, marital status, and years of work experience, as well as health-related behaviors linked to cannabis and MC among Cypriot nurses and midwifes.

## Methods

### Design, setting, and participants

A descriptive cross-sectional study with internal comparisons was performed during the 26^th^ Nurses and Midwives Congress in Cyprus, which took place on November 28–30, 2019, in Limassol. In this study convenience method was applied. All active registered nurses whom participate in 26^th^ Nurses and Midwives Congress in Cyprus, were eligible to participate, regardless of age, gender, and nationality. Final data population included 232 nurses and midwives from the private and public sectors.

### Data collection

Participation in the study was voluntary and anonymous in order to guarantee confidentiality. Questionnaires and consent forms were distributed to the nurses and midwives at the beginning of the conference. Then, after a short briefing on the study aim and procedures, nurses and midwives who wished to participate could place their filled-in questionnaires in sealed envelopes in a collection box located outside of the conference room.

Our study was approved by the National Bioethics Committee (Ref. No 2019.01.155) as well as the board of directors of the Cyprus Nurses and Midwifes Association, who organized the event.

### Instrument

The data collection instrument was the Attitudes, Beliefs, and Knowledge toward Medical Cannabis Questionnaire (MCQ) [13, 14], which was developed for cross-national studies on MC education among healthcare professionals and students [10, 14–17]. Thirteen questionnaire items assess attitudes and beliefs toward MC/cannabis (e.g., benefits, risks, effectiveness). Eighteen items assess beliefs and knowledge about MC effectiveness for medical conditions, while two items assess beliefs and attitudes regarding MC education. Educational training-related attitudes toward MC are assessed by two items with predefined answers. One item assesses participant’s attitudes toward formal and informal sources of information on MC. The MCQ has a high level of internal consistency (Cronbach’s alpha values ranging from 0.767 to 0.831) [13].

The MCQ was translated into Greek by Sokratous et al. [10]. The scale was translated from English to Greek by two independent translators familiar with Cypriot culture. The new Greek version of the instrument was compared with the previous one to generate a single reconciled version, which was then translated back into English. The final version of the instrument was pre-tested in a pilot study with 100 students in order to assess its readability and general comprehensibility. The metric properties of the scale were also tested in our previous study [10]. In this previous study, we used the MCQ to examine MC attitudes, beliefs, and knowledge among Greek-Cypriot nursing students and found that it exhibited a high level of internal consistency (Cronbach’s alpha values ranging from 0.75 to 0.85) [10].The authors in the present study re-examined the reliability of the MCQ; the result showed that MCQ exhibited a high level of internal consistency (Cronbach’s alpha value of 0.85).

The data collection instrument also included a section with variables on demographics characteristics (age, gender, religion, origin, family status, employment status), educational status level (years of study, highest degree completed, field of expertise, years’ work experience) and cannabis/MC-related behaviors.

### Data analysis

Descriptive statistics were calculated for the socio-demographic characteristics and MCQ items and expressed, as appropriate, as frequencies, mean values, or standard deviation. Responses to the ordinal MCQ variables were grouped into three categories: (a) agree/effective, (b) disagree/ineffective, and (c) do not know. Differences between the groups were assessed according to gender, age, religion, family status, and years of work experience work experience, and the Pearson Chi-square test was used accordingly. The SPSS version 25.0 statistical software was used for the data analysis. The significance level was set at α = 0.05.

## Results

### Socio-demographic characteristics

The final sample population consisted of 232 nurses and midwives (response rate of 46.4%). In total, 67(28.9%) participants were male, and 165(71.1%) were female. The mean age of the participants was 32.3 years old (SD: 9.2; range: 21–70). The vast majority of the nurses and midwives were Cypriot origin (*n* = 202, 87.1%), 12 (5.2%) were Greek, and 18 (7.7%) were foreigners. In addition, most participants were Christian Orthodox (*n* = 221, 95.3%) and the rest of them reported others ethnicities (*n* = 11. 4.7%). Concerning the employment status, the vast majority of the participants were employed (*n* = 220, 94.8%), and almost half of the sample, were working in government nursing positions (*n* = 104, 44.8%).

Finally, 164 (72.9%) participants held an undergraduate degree, 55 (23.7%) held a master’s degree and 9 (1.7%) held a PhD degree. Most participants had more than five years of work experience. In total, 184 (79.2%) participants were general practitioner nurses, 28 (12.1%) were midwives and 20 (8.7%) were mental health nurses (Table 1).

**Table 1:** The socio-demographics characteristics of the sample (*n* = 232)

Variables	n (%)
**Mean age** = 32.30 (Range 21—70 years / SD = 9.225)	
**Gender**	
Female	165 (71.1)
Male	67(28.9)
**Religious**	
Christian	221 (95.3)
Muslim	1 (0.4)
Non-denominational / Atheist	6 (2.6)
Other	4(1.7)
**Degree of loyalty**	
Not religious	19 (8.2)
Somewhat religious	77 (33.2)
Religious / Very religious	136 (58.6)
**Mother born**	
Cyprus	202 (86.8)
Greece	12 (5.3)
Other European Countries	5 (2.2)
Soviet Union	7 (3.1)
Middle Eastern country	1 (0.4)
South America	1 (0.4)
Other	4 (1.8)
**Family status**	
Single (not in a relationship)	51 (21.9)
Single (in a relationship)	62 (26.8)
Married / Civil partnership	105 (45.6)
Widow / Widower	1 (0.4)
Separated	1 (0.4)
Divorced	12 (4.9)
**Government position nurse**	
Yes	104 (44.8)
No	128 (55.2)
**Current employment status**	
Full-time / Part-time employed	220 (94.8)
Unemployed	12 (5.2)
**Academic status**	
Undergraduate degree (BA, BS)	164 (72.9)
Master degree (MA, MS)	55 (23.7)
PhD	9 (1.7)
Other post graduate level degree	4 (1.7)
**Work experience**	
0	11 (4.4)
1–2	37(15.8)
3–5	69 (29.8)
> 5 years	115 (50)
**Study filed**	
Nurse	184 (79.2)
Midwife	28 (12.1)
Mental health nurse	20 (8.7)

### Health-related behaviors linked to cannabis and MC among nurses and midwives and their relatives and friends

The vast majority of participants (*n* = 227, 97.8%) had never used prescribed MC, while 4 (1.8%) rarely used prescribed MC for personal purposes, and 1 (0.4%) used prescribed MC on a monthly basis for personal purposes. Moreover, participants 191 (82.3%) have never used marijuana for recreational purpose, while of the sample 31 (13.4%) used recreational marijuana every month, 6 (2.6%) people used recreational marijuana on a monthly basis and 4 (1.7%) participants use recreational marijuana on a weekly basis. Furthermore, 18 (7.8%) participants had a family member who use or used MC, while 13 (5.6%) participants had a family member who uses or had used recreational marijuana on a daily or weekly basis. Additionally, 28 (12.1%) participants of the sample had a friend or friends who uses or had used MC, while 83 (35.8%) had a friend or friends who uses or had used recreational marijuana on a daily or weekly basis.

### Nurses and midwives’ attitudes and beliefs about cannabis and MC

The majority of the participants strongly agreed with the idea that health and healthcare professionals should have formal training on MC before recommending to the patients (*n* = 133, 60.5%). Some participants believed that educational training on MC use should be integrated into the practice/clinical practice requirements of nurses (*n* = 77, 34.8%). Additionally, 107(46.1%) participants stated that educational training for MC must be integrated into academic programs for health and welfare professionals and 97 (42.2%) supported the notion that marijuana can be addictive. Many participants strongly agreed that additional research regarding MC use should be encouraged (*n* = 134, 58%), and more than half supported the idea that medical professionals who prescribe MC should have ongoing contact with their patients/clients (*n* = 119, 51.7%). Nearly one-third of the participants believed that, physicians should recommend MC as a form of medical therapy (*n* = 74, 31.9%), and a similar number would recommend MC use to the patients if they could do it (*n* = 86, 37.2%).

Furthermore, 74 (32.3%) participants somewhat agreed with the idea that using marijuana poses serious mental health risks, and 62 (27%) were aware of the benefits of MC. Meanwhile, almost the half of participants agreed that MC has significant physical health benefits, and one third of them, agreed that MC has significant mental health benefits. However, 80 (34.6%) participants strongly disagreed that marijuana should be legalized for recreational use.

A huge number of nurses and midwives reported a lack of knowledge of MC effectiveness for the majority of disorders included in the questionnaire. Nevertheless, some participants considered MC use to be acceptable for persistent muscle spasms (*n* = 67, 28.9%), insomnia/sleeping disorders (*n* = 62, 26.7%), and mental health conditions (*n* = 65, 28%), and strongly agreed that MC use is acceptable for terminal illnesses (*n* = 48, 20.3%).

### Nurses and midwives’ attitudes and beliefs about formal MC education

In total, 94 (41%) of the participants were neutral on answering to patient/client questions about MC, while 128 (55.9%) of them strongly agreed. However, 66 (28.8%) believed that nurses should receive formal education about medical marijuana laws and regulations.

### Nurses and midwives’ sources of information about MC

Concerning nurses and midwives’ sources of information about MC*,* 197 (84.9%) participants had never received any formal education about MC. Furthermore, one third of nurses and midwives reported that, during their studies should receive formal education about MC in class (*n* = 73, 31.6%) or in clinical practice (*n* = 26, 11.2%). More of the half of them, (*n* = 121, 52.2%) believed that formal education about MC in class and in clinical practice is a must. Finally, the most frequently reported sources of information were medical literature (*n* = 146, 62.9%), physicians (*n* = 97, 42%), and experiences with patients/clients (*n* = 97, 441.8%).

### Socio-demographic characteristics about MC attitudes, beliefs, and knowledge of nurses and midwives

Gender had a statistically significant positive effect on participants’ attitudes and beliefs about MC. Male participants expressed in strong percentages their positive preference to use cannabis for recreational purposes (*p* < 0.01, 26.8% vs. 13.4%) compared with female group. Male participants expressed in strong percentages that healthcare professionals should have formal training on MC before recommending it to a patient compared with female group (*p* < 0.05, 94% vs. 87.9%). However, a larger number of female participants agreed that healthcare professionals who prescribe MC should have ongoing contact with their patients compared with male group (*p* < 0.05, 96.7% vs. 85%). Furthermore, more than twice as many males compared with females’ participants group, believed that they were prepared to answer patients’ questions about MC (*p* < 0.01, 41.8% vs. 18.8%) (Table 2).

**Table 2:** Attitudes, beliefs and knowledge about medical cannabis among nurses and midwives in Cyprus (*n* = 232)

	**Gender % (n)**									
	**Total (*n* = 232)**		**Male** **(*n* = 67)**			**Female** **(*n* = 165)**				***P* value**
^b^**Part A: Health-related behaviors linked to cannabis and medical cannabis**										
Personal use of cannabis for recreational purposes	17.2(40)		26.8(18)			13.4 (22)				**
Friends who use cannabis for recreational purposes	33.2(83)		44.8(30)			32.1(53)				*
^a^**Part B: Attitudes & beliefs on cannabis and medical cannabis (MC)**										
Healthcare professionals should have formal training about MC before recommending it to a patient	89.7(208)		94(63)			87.9(145)				*
Healthcare professionals who prescribe MC should have ongoing contact with their patients	93.5(217)		85(57)			96.7(160)				*
^a^**Part C: Attitudes & beliefs on medical cannabis education (MC)**										
I am prepared to answer patient’s questions about MC	25.4(59)		41.8(28)			18.8(31)				**
**Part D: Sources of information about medical cannabis (MC)**										
Personal use	4.7(11)		5.6(4)			4.2(7)				*
	**Age Group % (n)**									
	**Total** **(*n* = 232)**		**20–30 years old(*n* = 144)**		**31–40 years old(*n* = 43)**			≥ **41 years old(*n* = 45)**		***P* value**
^a^**Part B: Attitudes & beliefs on cannabis and medical cannabis (MC)**										
Additional research regarding MC use should be encouraged	94.4(219)		98.6(142)		88.4(38)			86.7(39)		*
^a^**Part C: Attitudes & beliefs on medical cannabis education (MC)**										
Educational training for MC must be integrated into academic programs	98.7(229)		100(144)		95.2(40)			100(45)		*
	**Religion % (n)**									
	**Total** **n(232)**	**Christian Orthodox** **(*n* = 221)**		**Muslim** **(*n* = 1)**		**Non denomination / Atheist(*n* = 6)**			**Other** **(*n* = 4)**	***P* value**
^a^**Part A: Health-related behaviors linked to cannabis and medical cannabis**										
Friends who use any cannabis	12.1(28)	10.1(24)		-		33.3(2)			50(2)	*
^a^**Part B: Attitudes & beliefs on cannabis and medical cannabis (MC)**										
Educational training for MC must be integrated into clinical practice	84.1(195)	83.7(185)		-		100(6)			100(4)	*
Additional research regarding MC use should be encouraged	94(218)	95.4(211)		100(1)		50(3)			75(3)	*
	**Family Status % (n)**									
	**Total** **(*n* = 232)**	**Married** **(*n* = 105)**					**Unmarried** **(*n* = 127)**			***P* value**
^b^**Part A: Health-related behaviors linked to cannabis and medical cannabis**										
Personal use of cannabis for recreational purposes	17.7(41)	11.4(12)					22.8(29)			*
Friends who use cannabis for recreational purposes	35.6(83)	27.6(29)					42.5(54)			*
^a^**Part B: Attitudes & beliefs on cannabis and medical cannabis (MC)**										
Using cannabis poses serious mental health risks	72(167)	66.8(68)					77.9(99)			*
^b^**Part E: Sources of information about medical cannabis (MC)**										
Other nurses	25(58)	17.1(18)					31.5(40)			*

^*^p < .05; **p < .01; ***p < .001

^a^The table presents the n and % of participants who answered that they ‘agree’

^b^The table presents the n and % of participants who answered that ‘yes’

Moreover, concerning the age of year-old group, the vast majority of the sample agreed that, additional research about MC use must be integrated into academic programs (98.6% in the age of 20–30-year-old group, 88.4% of the 31–40-year-old group and 86.7% of the ≥ 41-year-old group, *p* < 0.05), while almost all of them (95.2%) agreed that educational training for MC must be integrated into academic programs (*p* < 0.05).

Furthermore, concerning the relational status, all participants included in this study (non-denominational/atheist, Christian Orthodox, Muslim), agreed that educational training about MC must be integrated (*p* < 0.05), while Muslim participants agreed that, additional research about MC use should be encouraged (*p* < 0.05).

Finally, concerning the marital status, unmarried participants expressed their positive preference about cannabis use for recreational purposes in twice as strong, compared with married participants group (*p* < 0.01, 22.8%Vs 11.4%). Additionally, almost the half of unmarried participants (42.5%) reported that, had friends who use cannabis for recreational purposes, compared with married participants group, whom reports less percentages (27.6%, *p* < 0.05). Unmarried participants agreed that using cannabis might develop serious mental health risks compared with married participants group (*p* < 0.05, 77.9% vs. 66.8%).

There was no statistically significant difference between work years of experience and attitudes, knowledge, and beliefs about medical cannabis among nurses and midwives in Cyprus (Table 2).

### Socio-demographic characteristics about MC attitudes, beliefs, and knowledge of nurses and midwives for the treatment of specific disorders

Male participants, expressed in strong percentages their positive consideration about MC use to be acceptable for the treatment of anorexia, HIV/AIDS, inflammatory bowel disease (e.g., Crohn’s disease), nausea, and persistent muscle spasms, compared with female participants group (*p* < 0.05). Additionally, percentages of the participants in the age of 20–30-year-old group, believed that MC to be acceptable for the treatment of nausea and/or vomiting due to cancer treatment, HIV/AIDS, mental health conditions (e.g., PTSD, depression, anxiety), MS, Parkinson’s disease, and terminal illnesses, compared with those participants in other age groups (*p* < 0.05). Participants in the age of 31–40-year-old group, considered MC to be acceptable for the treatment of eating disorders, insomnia/sleep disorders and nausea, compared with other groups. The ≥ 41-year-old age group expressed their positive believed as strong, that MC use is acceptable for the treatment of seizures and epilepsy compared with other groups (*p* < 0.05). Finally, non-denominational and atheist participants groups, expressed their positive believed as strong and considered MC to be acceptable for the treatment of Alzheimer’s disease, eating disorders (e.g., anorexia), and mental health conditions (e.g., PTSD, depression, anxiety) compared with other groups (*p* < 0.050).

Finally, participants with years of work experience, expressed in strong percentages their positive consideration about MC use to be acceptable for the treatment of nausea and/or vomiting due to cancer treatment and/or terminal illness compared with other groups (*p* < 0.05**,** Table 3).

**Table 3:** Nurses’ knowledge about MC in the treatment of specific disorders (*n* = 232)

	**Gender % (n)**										
	**Total** **(*n* = 232)**		**Male** **(*n* = 67)**			**Female** **(*n* = 165)**					***P* value**
Anorexia	19(44)		50(22)			50(22)					*
HIV/AIDS	24.1(56)		33.9(19)			66.1(37)					**
Inflammatory bowel disease (eg. Crohn’s disease)	44.5(94)		31.9(30)			68.1(64)					*
Nausea	27.6(64)		39.1(25)			60.1(39)					*
Persistent muscle spasm	59.9(139)		28.8(40)			71.2(99)					*
	**Age % (n)**										
	**Total** **(*n* = 232)**		**20–30 years old (*n* = 144)**		**31–40 years old (*n* = 43)**			** > 41 years old (*n* = 44)**			***P* value**
Nausea and/or vomiting due to cancer treatment	49.6(115)		53.5(77)		44.2(19)			43.2(19)			**
Eating disorders (eg. Anorexia)	31.9(74)		30.6(44)		34.8(15)			34.1(15)			**
HIV/AIDS	23.7(55)		25(36)		23.2(10)			20.5(9)			*
Insomnia / sleep disorders	59.1(137)		59(85)		60.5(26)			59.1(26)			*
Mental health conditions (eg. PTSD, depression, anxiety,etc.)	66.4(154)		70.1(101)		55.8(24)			65.9(29)			*
Multiple Sclerosis	53.9(125)		58.3(84)		46.5(20)			47.7(21)			*
Nausea	27.6(64)		23.6(34)		38.9(15)			34.1(15)			***
Parkinson’s disease	40.9(95)		42.4(61)		37.2(16)			40.9(18)			*
Seizure / Epilepsy	47.4(110)		47.9(69)		44.2(19)			50(22)			*
Terminal illness	72.8(169)		80.6(116)		69.8(30)			59.1(26)			**
	**Religion % (n)**										
	**Total** **(*n* = 232)**	**Christian Orthodox** **(*n* = 221)**		**Muslim** **(*n* = 1)**		**Non denomination / Atheist** **(*n* = 6)**				**Other** **(*n* = 4)**	***P* value**
Alzheimer’s disease	42.7(99)	41.2(91)		-		6.1(6)				2(2)	*
Eating disorders (eg. Anorexia)	31.9(74)	98.6(73)		-		1.3(1)				-	*
Mental Health conditions (eg. Depression, anxiety, etc.)	66.8(155)	95.5(148)		-		3.9(6)				0.6(1)	**
	**Work Experience % (n)**										
	**Total** **(*n* = 232)**		**0 years** **(*n* = 11)**	**1–2 years** **(*n* = 37)**			**3–5 years** **(*n* = 69)**		** > 5 years** **(*n* = 115)**		***P* value**
Nausea and/or vomiting due to cancer treatment	49.6(115)		45.5(5)	37.8(14)			55.1(38)		50.4(58)		*
Terminal illness	74.1(172)		63.6(7)	72.8(27)			88.4(61)		67(77)		*

^*^p < .05; **p < .01; ***p < .001

The table presents the n and % of participants who answered that they ‘agree’

## Discussion

Studying the national and international literature, it seems that, this study is the first attempt to record and evaluate the attitudes, beliefs and knowledge of Cypriot nurses and midwives. Given the relatively high response rate and convenience sampling, it is possible to generalize the present study’s findings to the entire nursing and midwifery population in Cyprus.

The vast majority of previous studies about MC use, have focused on prescribers or a single disease category [6, 11, 18, 19]. Our findings focus on attitudes, beliefs, and knowledge of Cypriot nurses and midwives. The present study adds more evidence to the existing literature providing new data on the attitudes, beliefs, and knowledge among health care professionals.

Our results seem to be rather recommending in favor of MC use. The main findings of the present study showed that, Cypriot nurses and midwives reported lack of knowledge regarding the risks and benefits about MC use to patients. The participants reported lack of confidence when they discussed MC benefits for specific disorders with their patients. However, specific number of participants believed MC use was considered acceptable for the patients with persistent muscle spasms, insomnia/sleeping disorders, mental health conditions, and terminal illnesses.

Our results showed that, the majority of the participants believed that formal training on MC should be integrated into academic programs. Cypriot nurses and midwives expressed the necessity of urgent training under the current curriculum. Specifically, more than the half of participants believed that it is necessary to have formal training about MC use before recommending to their patients. The participants supported that, educational training programs about MC use should be integrated into the practice/clinical practice. Other similar studies associating MC knowledge indicators with relevant attitudes of healthcare professionals in the United States (US), have shown that nurses have higher self-perceived knowledge and more positive attitudes toward MC than other healthcare professionals [20].However, a lack of formal education and training and significant knowledge gaps regarding MC among nurses and physicians have been reported [21]. Furthermore, since MC laws about use is rapidly changing in some countries, such as the US, advanced practice registered nurses during their clinical practice have been authorized to recommend MC use to their patients [22].

Comparing our findings with the results of our previous study [10], both studies presented similarities. Specifically, the majority of the Cypriot nursing students in previous study, believed that formal training on MC should be integrated into academic programs and expressed the necessity of urgent training about MC under the current curriculum. Our results are on the same line with others studies worldwide [20, 21].

Additionally, in the present study nurses and midwives strongly supported that, educational training programs on MC use should be integrated into the practice/clinical practice. Our results supported from the relevant literature. Research evidences supported that, health practitioners have insufficient theoretical and clinical knowledge about MC use and benefits [20, 21]; these specific knowledges important and necessary to increase their competency about the benefits of MC use [21].

In the present study, concerning the socio-demographic characteristics of the participants, we examined the association among gender, age, religion, and years of work experiences in the clinical sectors and attitudes, beliefs, and knowledge about MC use of the sample. As a result, the present study adds more evidence to the existing literature concerning the socio-demographic characteristics of the health care professionals. Our results pointed out that, gender had a statistically significant positive effect on participants’ attitudes and beliefs about MC. More specifically, male participants reported higher frequency about cannabis use for recreational purposes, compared with female group. Regarding reported higher frequency about cannabis use for recreational purposes amongst male participants, this might a result of low and undervalued social stigma amongst males who use cannabis for recreational purposes [2, 3],compared with females. Generally, a higher frequency about cannabis use for recreational purposes amongst male participants, has been associated with socio-cultural explanations, including factors related to gender role, as well as with biological and psychological parameters. The impact of personality traits also involved. Males, might express themselves more easily and may not be afraid of being cultural stigmatized by their society. However, evidences from the international literature concerning the gender, showed contradictory results [23, 24].

Concerning the patients, a larger number of female participants in this study, agreed that healthcare professionals who prescribe MC should have ongoing contact with their patients compared with male group. On the other hand, more than twice as many males compared with females’ participants group, believed that they were prepared to answer patients’ questions about MC. Male participants expressed in strong percentages that healthcare professionals should have formal training on MC before recommending it to a patient compared with female group. In this study, our results showed that, the vast majority of the sample (male and female), agreed that, additional research about MC use must be integrated into academic programs, while almost all of them agreed that educational training for MC must be integrated into academic programs.

Moreover, concerning the age of year-old groups among the participants, our results showed similarities among these groups. The vast majority of the sample in all groups, strongly agreed that additional research about MC use must be integrated into academic programs; almost all of them pointed out that educational training for MC must be integrated into academic programs.

Furthermore, concerning the relational status, our results showed that all participants included in this study, agreed that educational training about MC must be integrated and additional research about MC use should be encouraged.

Additionally, the marital status, the study showed that, the unmarried participants expressed their positive preference about cannabis use for recreational purposes in twice as strong, compared with married participants group. Concerning the social transactions among married and unmarried participants, almost the half of unmarried participants reported that, had friends who use cannabis for recreational purposes, compared with married participants group, whom reports less percentages. However, although they had friends who use cannabis for recreational purposes, unmarried participants agreed that using cannabis might develop serious permanent mental health symptoms/diseases. Their answers may arise from their attitudes, knowledge, and beliefs about cannabis for recreational purposes as healthcare professionals.

Although that there is no statistically significant difference between years of work experiences in the clinical sectors and MC use among nurses and midwives in Cyprus, it seems that there is a positive trend about years of work experiences and MC among nurses and midwives in Cyprus. Nurses and midwives in Cyprus having more years of work experiences in the clinical sectors, might have a more positive attitudes concerning MC use. Similar results found among other professionals worldwide concerning MC use [14].

In this study, our results seem to be rather recommending in favor of MC use; additionally, a small number of nurses and midwives supported the legalization of marijuana for recreational use. The vast majority of the participants in the present study would recommend MC to their patients if it were legalized. However, the majority of the sample population strongly disagreed with the legalization of marijuana for recreational use. This result is in contrast with our previous study of Cypriot nursing students, in which the third-year students were more in favor of legalizing marijuana for recreational use [11]. Low rates of support for the legalization of marijuana for recreational use were also observed in a US study of healthcare professionals [19, 25]. Although, the literature has shown that healthcare professionals support MC use and its legalization [22, 26–29]. In our study, most of the participants seemed to have lack of basic knowledge of the content, effects, and legality of MC. These results confirm prior reports of significant gaps in the MC knowledge of healthcare professionals [19, 30]. Since the nuances of developing MC education frame are critical, Cypriot nurses and midwives need to be educated about the advantages and disadvantages of MC use for specific disorders.

Finally, the above findings need to be viewed in the context of certain methodological limitations. The data collection took place during the 26^th^ Nurses and Midwives Congress in Cyprus, which took place on November 28–30, 2019, in Limassol. In the 26^th^ Nurses and Midwives Congress, hence, nurses and midwives that were absent on that days were excluded, along with those who refused to participate. As a result, the observed results may be underestimated or might be were slightly different. Additional limitations include lack of triangulation with qualitative data, as well as possible underestimation of the actual frequency of cannabis use, or positive attitudes towards MC. This may be attributed to one’s need to preserve a positive personal image, thus avoiding to report adverse behaviours (i.e. illegal substance use or socially unpopular attitudes).

More importantly, the cross-sectional nature of the study does not permit any inference with regard to the direction of the observed association between nurses and midwives’ attitudes, beliefs and knowledge and medical cannabis used. Nevertheless, the large sample and the use of more appropriate and robust instrument in the present study allow for a more accurate estimation of the occurrence of nurses and midwives’ attitudes, beliefs and knowledge and medical cannabis used.

## Conclusions

The conclusions seem to be rather recommending in favor of MC use**.** The main findings of the present study were that, the vast majority of Cypriot nurses and midwives strongly believed that more MC education is needed in order to address nurses and midwives’ lack of knowledge about MC benefits for specific disorders, a substantial divergence between their current and desired levels of knowledge, and their extensive lack of knowledge of the risks and benefits of MC use by patients. Additionally, Cypriot nurses and midwives reported lack of knowledge and absence of guidelines and protocols on clinical health sectors about MC use. Participants proposed enriching nursing curricula with theoretical and clinical/laboratory courses on MC during studies and clinical practice. Additional tailoring interventions should be established to decrease recreational cannabis use among Cypriot nurses and midwives.

## Data Availability

The datasets generated and/or analyzed during the present study are not publicly available because the authors are currently working on them in order to prepare the final version of this manuscript. However, they are available from the corresponding author upon reasonable request.
